# Analysis of mouse intestinal organoid culture with conditioned media isolated from mucosal enteric glial cells

**DOI:** 10.1016/j.xpro.2022.101351

**Published:** 2022-04-28

**Authors:** Meryem B. Baghdadi, Tae-Hee Kim

**Affiliations:** 1 Program in Developmental & Stem Cell Biology, The Hospital for Sick Children, Toronto, ON M5G 0A4, Canada; 2 Department of Molecular Genetics, University of Toronto, Toronto, ON M5S 1A8, Canada

**Keywords:** Cell Biology, Cell culture, Cell isolation, Flow Cytometry/Mass Cytometry, Microbiology, Molecular Biology, Gene Expression, Stem Cells, Organoids

## Abstract

This protocol describes the isolation and culture of 3D intestinal crypt organoids with stromal niche cells. We show a murine organoid culture system that utilizes conditioned media isolated from primary, mucosal enteric glial cell culture. We describe three assays of analyzing this organoid culture: flow cytometry, gene expression, and organoid morphology analyses. This protocol can also be used to study the mechanisms of stem cell interaction with other stromal niche cell types such as mesenchymal cells and innate immune cells.

For complete details on the use and execution of this protocol, please refer to Baghdadi et al. (2021).

## Highlights

• Organoid protocol to study intestinal stem cell interactions with mucosal glial cells
• Optimized protocol to isolate mucosal glial cells and collect their conditioned media
• Efficient analysis of organoid morphology, gene expression and cellular composition

## Graphical abstract

**Figure:**
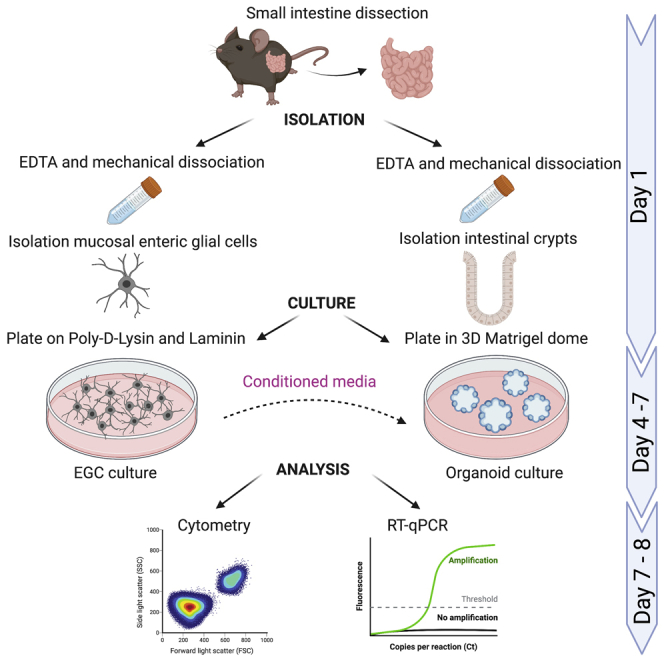

## Before you begin

### Ethical biomedical research

All procedures involving experimental animals should be approved by relevant national and institutional animal committee members in accordance with their guidelines. Animal housing, husbandry and handling were approved and performed in accordance with the Animals for Research Act of Ontario and the Guidelines of the Canadian Council on Animal Care at The Toronto Centre for Phenogenomics (TCP).

### Prepare reagents and materials

1. At least one day before the experiment.
  a. Reconstitute recombinant proteins according to manufacturers’ recommendations and store aliquots at −20°C.
  b. Thaw Matrigel bottle overnight on ice at 4°C (fridge or cold-room). Make 1 mL aliquot in 1.5 mL microcentrifuge tubes on ice. Store at −20°C.
2. The day of the experiment.
  a. Thaw one aliquot of Matrigel on ice 1 h before use.
  b. Prepare the buffers and media, as described in the “materials and equipment” section.
  c. Pre-warm ENR (EGF/Noggin/R-Spondin) media at 37°C.
  d. Set benchtop centrifuges to 4°C.
  e. Place a 24-well plate in the CO_2_ incubator at 37°C.

### Coat culture plate for enteric glial cell (EGC) culture

**Timing: 3 h**

**CRITICAL:** Coating the culture plate should be done in a sterile culture laminar flow hood.

3. Dilute Poly-D-lysine (PDL) stock (1 mg/mL) at 1:10 with sterile water to a final concentration of 100 μg/mL.
4. Coat three wells of a 12-well plate with 1 mL of PDL for at least 1 h at room temperature (20°C–22°C).
5. Remove the PDL with a sterile pipette.
6. Allow the plates to air dry completely under the culture hood.
7. Dilute the laminin stock (0.5 mg/mL) at 1:50 with sterile PBS 1× to a final concentration of 10 μg/mL.
8. Add 500 μL of diluted laminin to the wells containing dried PDL.
9. Incubate the coated plates at 37°C for 2 h (quick coating).

***Note:*** Laminin coating can also be done overnight (12–16 h) at 4°C (slow coating). Both quick and slow coating methods allow equivalent EGC adhesion.

10. Remove the laminin solution carefully with a pipette to avoid scratching the surface. Gently wash the wells three times with sterile PBS 1×.
11. Add complete EGC culture media to each well and maintain the plates at 37°C before adding the EGC suspension.

***Note:*** Coated plates can be prepared ahead and stored at 4°C for up to 3 days. After washing the laminin (step 8 above), add 2 mL of sterile PBS 1× per well, seal the plate with parafilm and store at 4°C.

## Key resources table

**Table:** 

REAGENT or RESOURCE	SOURCE	IDENTIFIER
**Antibodies**		

CD24-Pacific Blue (1/500)	BioLegend	Cat # 01829
Epcam-APC (1/500)	Abcam	Cat # ab95641
Rabbit anti-GFAP (1/200)	DAKO	Cat # Z0334
Alexa fluor 488 goat anti-rabbit (1/500)	Thermo Fisher Scientific	Cat # A-11078

**Chemicals, peptides, and recombinant proteins**		

Propidium iodide (1/1000)	Invitrogen	Cat # P1304MP
Matrigel	Corning	Cat # CACB356231
EGF	Sigma-Aldrich	Cat # E9644
Noggin	Cedarlane labs	Cat # 6057NG
R-spondin-1	RnD	Cat # 3474RS
GDNF	PeproTech	Cat # 450-44
Cell Recovery Solution	Corning	Cat # C354253
TrypLE express	Thermo Fisher Scientific	Cat # 12604013
DMEM-F12	Thermo Fisher Scientific	Cat # 21331020
N-2 Supplement	Thermo Fisher Scientific	Cat # 17502-048
B-27 Supplement, serum free	Thermo Fisher Scientific	Cat # 17504-044
GlutaMAX	Thermo Fisher Scientific	Cat # 35050-038
HEPES (1 M)	Thermo Fisher Scientific	Cat # 15630-056
N-Acetyl-L-cysteine	Thermo Fisher Scientific	Cat # A9165
Penicillin-Streptomycin	Thermo Fisher Scientific	Cat # 15140130
Poly-D-Lysine	Sigma-Aldrich	Cat # P6407-5MG
Laminin	Invitrogen	Cat # 23017015
EDTA	Invitrogen	Cat # 15575020
Gentamicin	Thermo Fisher Scientific	Cat # 15750060
Hanks’ Balanced Salt Solution (HBSS)	Thermo Fisher Scientific	Cat # 14175095
Sucrose	Sigma-Aldrich	Cat # S8501
Sorbitol	Sigma-Aldrich	Cat # S1876
PBS 1× (no calcium, no magnesium)	Thermo Fisher Scientific	Cat # 10010023
Fetal Bovine Serum (FBS)	Thermo Fisher Scientific	Cat # 26140087
Hoechst 33342 (1/5000)	Thermo Fisher Scientific	Cat # 1799639

**Critical commercial assays**		

SuperScript® III enzyme	Invitrogen	Cat # 18080093
RNeasy® Plus Mini Kit	QIAGEN	Cat # 741344
RNeasy® Plus Micro Kit	QIAGEN	Cat # 74034
Power SYBR green master mix	Applied Biosystems	Cat # 4368706

**Experimental models: Organisms/strains**		

*Lgr5-eGFP* mouse line: adult (6–10 weeks), male or female	The corresponding lab	Barker et al. (2007)

**Oligonucleotides**		

RT-qPCR primers	Baghdadi et al. (2021)	Table 1

**Software and algorithms**		

FlowJo	BD Biosciences	https://www.flowjo.com
Prism	Prism	http://www.graphpad.com
ImageJ	Schneider et al. (2012)	https://imagej.nih.gov/ij/download.html

**Others**		

Clear polystyrene sterile certified non-pyrogenic 12-well plate	VWR	Cat # 10062-894
Clear polystyrene sterile certified non-pyrogenic 24-well plate	VWR	Cat # 10062-896
0.22 μm pore size syringe filter	TPP	Cat # 99722
1 mL syringe Plastipak	BD	Cat # 305959
15 mL conical tube Falcon	BD	Cat # 352097
50 mL conical tube Falcon	BD	Cat # 352098
RNase Away	Thermo Fisher Scientific	Cat # 7002
Flow cytometer	No recommended vendor	N/A
Nanodrop photospectrometer	Thermo Fisher Scientific	Cat # ND-2000

**Table 1:** List of RT-qPCR primers to detect specific crypt cell type

Gene	Cell marker	Forward	Reverse	Amplicon size
*Lgr5*	Stem cells	GACAATGCTCTCACAGAC	GGAGTGGATTCTATTATTATGG	162 bp
*Olfm4*	Stem cells	GCCACTTTCCAATTTCAC	GAGCCTCTTCTCATACAC	199 bp
*Axin2*	Stem cells	GGACTGGGGAGCCTAAAGGT	AAGGAGGGACTCCATCTACGC	334 bp
*Lrig*	Stem cells	TTCCTTACCGGTGAGACTGG	CCATCACTGTGCCAACACTT	190 bp
*Lyz1*	Paneth cells	GGAATGGATGGCTACCGTGG	CATGCCACCCATGCTCGAAT	290 bp
*Defa5*	Paneth cells	TATCTCCTTTGGAGGCCAAG	TTTCTGCAGGTCCCAAAAAC	120 bp
*Ki67*	Transit amplifying cells	ATCATTGACCGCTCCTTTAGGT	GCTCGCCTTGATGGTTCCT	104 bp
*Tbp*	Normalization gene	ATCCCAAGCGATTTGCTG	CCTGTGCACACCATTTTTCC	91 bp
*Rpl13*	Normalization gene	GTGGTCCCTGCTGCTCTCAAG	CGATAGTGCATCTTGGCCTTTT	152 bp

## Materials and equipment

**Table:** Gut buffer

Reagent	Final concentration	Amount
Hank’s Balanced Salt Solution (HBSS)	n/a	48.5 mL
Fetal bovine serum (FBS)	2%	1 mL
HEPES (1 M)	10 mM	500 μL
**Total**	**n/a**	**50 mL**

***Note:*** Extra amount of buffer can be kept at 4°C for up to a month.

**Table:** Chelating solution

Reagent	Final concentration	Amount
Phosphate buffer saline (PBS) 1×	n/a	9.6 mL
EDTA (0.5 M)	20 mM	400 μL
**Total**	**n/a**	**10 mL**

***Note:*** Extra amount of buffer can be kept at 4°C for up to a month.

**Table:** Crypt dissociation buffer

Reagent	Final concentration	Amount
PBS 1×	n/a	100 mL
Sucrose	1.5%	1.5 g
Sorbitol	1%	1 g
**Total**	**n/a**	**100 mL**

***Note:*** Extra amount of buffer can be kept at 4°C for up to a month.

**Table:** ENR media

Reagent	Final concentration	Amount
DMEM-F12	n/a	46.9 mL
Glutamax (100×)	1%	500 μL
Penicillin-Streptomycin (100×)	1%	500 μL
HEPES (1 M)	10 mM	500 μL
B27 (50×)	1×	1 mL
N2 (100×)	1×	500 μL
NAC (N-acetyl-L-cysteine; 1 M)	1 μM	50 μL
Recombinant R-Spondin 1 (1 mg/mL)	1 μg/mL	5 μL
Recombinant Noggin (100 μg/mL)	100 ng/mL	10 μL
Recombinant EGF (500 mg/mL)	50 ng/mL	5 μL
**Total**	**n/a**	**50 mL**

***Note:*** This media can be stored at 4°C and used up to 1 week.

**Table:** EGC isolation solution

Reagent	Final concentration	Amount
PBS 1×	n/a	490 mL
EDTA (0.5 M)	5 mM	5 mL
HEPES (1 M)	10 mM	5 mL
**Total**	**n/a**	**500 mL**

***Note:*** Extra amount of buffer can be kept at 4°C for up to a month.

**Table:** EGC culture media

Reagent	Final concentration	Amount
DMEM/F12	n/a	44 mL
FBS	10%	5 mL
Penicillin -Streptomycin (100×)	1%	500 μL
Gentamicin (50 mg/mL)	20 μg/mL	20 μL
Glutamax (100×)	1%	500 μL
GDNF (10 μg/mL)	10 ng/mL	50 μL
**Total**	**n/a**	**50 mL**

***Note:*** This media can be stored at 4°C and used up to 1 week.

**CRITICAL:** All solutions should be filtered through 0.22 μm pore size filter before use and stored at 4°C.

## Step-by-step method details

### Part 1: Preparation of crypt organoid culture

**Timing: 3 h**

This protocol will allow you to establish crypt organoids from the murine small intestine. The resultant crypt suspension is mixed with Matrigel, and domes are deposited in a 24-well culture plate. After adding ENR (EGF, Noggin and R-Spondin) media, crypts develop into organoids, which contain *de novo* crypts and villus-like structures, recapitulating the *in vivo* intestine structure. For further information on the use and characterization of organoids, please refer to the previously published work (Sato et al., 2009; Mahe et al., 2013; Sato and Clevers, 2013).

#### Part 1A: Isolation of intestinal crypts

**CRITICAL:** To ensure minimal damage to the crypts, keep PBS 1×, Gut buffer, Chelating buffer and Crypt dissociation buffer on ice during the entire procedure. Have all the tools and reagents ready before sacrificing the mouse and perform the isolation on ice.

1. Euthanize mice using an authorized, legal method, approved by the institution where the research is conducted.
2. Sterilize the abdomen with 70% Ethanol.
3. Make an incision into the abdominal cavity from the external genitalia to the rib cage by cutting abdominal musculature on both sides.
  a. Lift the liver and cut the intestine from the stomach at the pyloric sphincter.
  b. Cut across the large intestine just prior to the anus to release the whole gut.
  c. Cut the mesenteric attachment of the intestine if necessary and gently pull the gut out of the abdominal cavity (Methods video S1A).
4. Place the gut in a petri dish filled with the ice-cold gut buffer.
5. Tear the mesentery to unfold the gut (Methods video S1A).
6. Separate the small intestine from the colon by cutting the gut at the ileocecal junction (Methods video S1A).
7. Flush the intestine of fecal content with the ice-cold gut buffer (Methods video S1B) using a 1 mL syringe mounted with a 1,000 μL tip. If necessary, use forceps to gently push the feces out of the gut.
8. Cut the intestine into 3 equal pieces (roughly corresponding to the duodenum, jejunum, and ileum).
9. Cut open each segment longitudinally so that the inside of the intestine is facing up.
10. Use a slide to gently scrape out the villi (Methods video S2). Discard the part of the intestinal lumen that remains on the slide.

**CRITICAL:** To avoid crypt loss, scrape the intestine gently.

11. Place the intestinal segments in a conical 50 mL tube filled with the 20 mL gut buffer and invert gently the tube 3 times. Discard the supernatant.
12. Recover the intestinal segments with forceps and cut them into 0.5 cm pieces. Transfer the pieces to a 15 mL tube containing 10 mL of ice-cold chelating buffer.
13. Shake the tube using either an orbital shaker or a rocking plate at 4°C (fridge or cold-room) for 25 min at 20 rpm.
14. Gently invert the tube and filter the solution through a 100 μm strainer into a 50 mL conical tube to separate the tissue pieces from the solution containing cellular debris.

***Note:*** We recommend keeping this solution until the end of the experiment. If the crypt isolation is successful, discard it. If the crypt isolation is not effective, check if the crypts have been lost in this suspension (see the “troubleshooting” section).

15. Use forceps to transfer the intestine pieces into a 50 mL conical tube containing the 25 mL ice-cold crypt dissociation buffer.
16. Orient the tube at 45° to the ground and shake it for 4–6 min with a gentle and regular motion (Methods video S3). Every 2 min, place the tube on ice to rest for 30 s.

**Figure 1:**
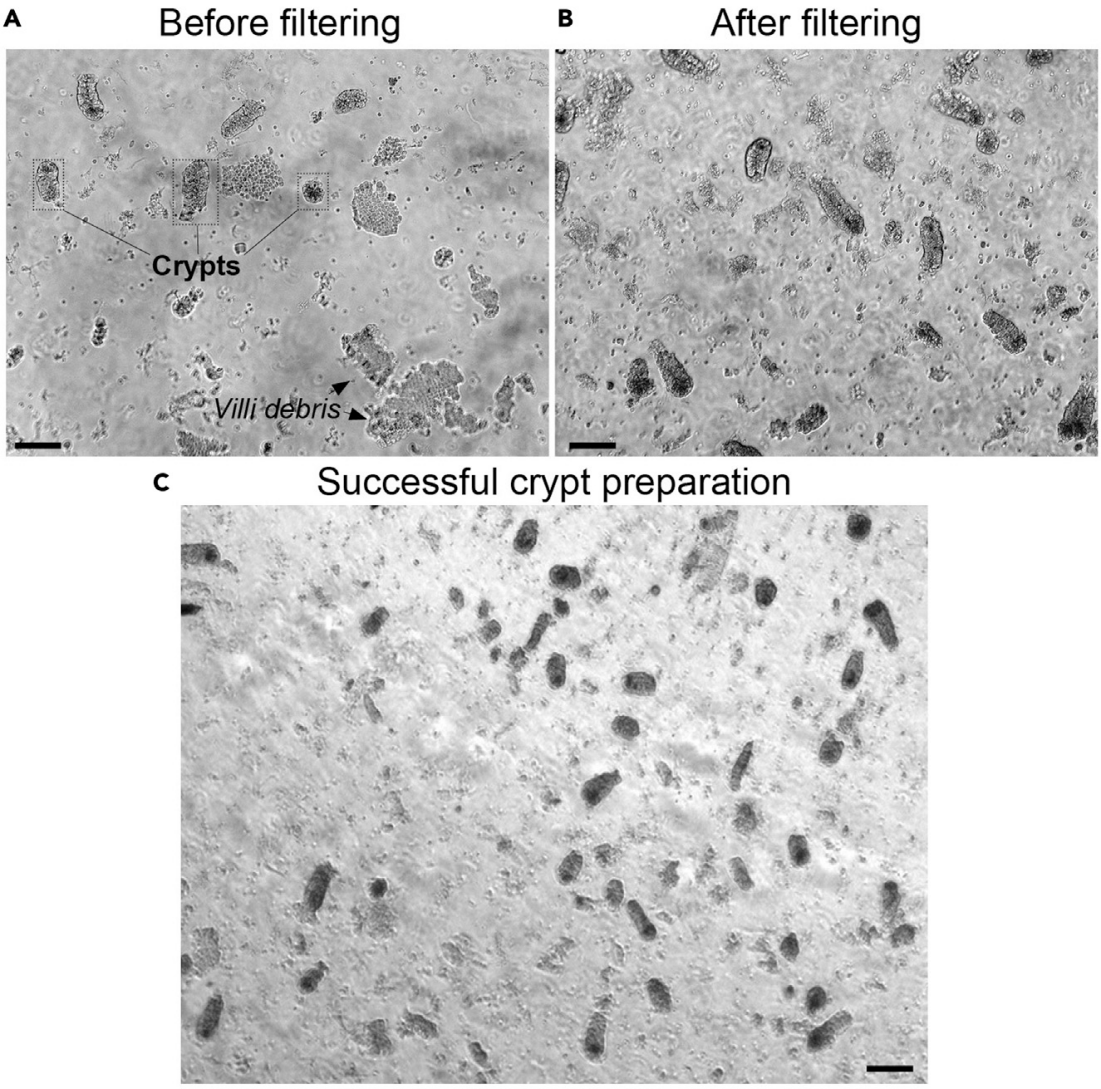
Crypt preparation at different steps of the protocol (A–C) (A) Step 16 (B) Step 18, and (C) Step 22. Scale bar, 50 μm.

***Note:*** Pipet 50 μL of the solution and check under the microscope for crypt enrichment. If necessary, shake the tube for 2 extra minutes, and check again (Figure 1A).

17. Place the tube on ice and allow the fragments settle down.
18. Filter the solution through a 70 μm filter into a 50 mL conical tube on ice (Figure 1B).

***Note:*** This step will remove villi and enrich for crypts.

19. Add 10 mL of the crypt dissociation buffer to wash the strainer. Discard the strainer.
20. Centrifuge the crypt-containing solution at 150 g for 10 min at 4°C.
21. Carefully discard the supernatant (single cells) and resuspend the pellet (crypts) in 10 mL of ice-cold PBS 1× (Figure 1C).
22. Count the number of crypts in 10 μL. The total number of crypts in the tube correspond to the number of crypts in 10 μL × 1,000.
23. Transfer the volume required for 300 crypts/well to a new 15 mL conical tube.

***Note:*** For a full 24-well plate: 300 crypts × 24 wells = 7,200 crypts.

***Note:*** If the number of crypts in 10 μL is too high and difficult to count, further dilute the crypt suspension by adding an extra 10 mL of ice-cold PBS 1X.

24. Centrifuge the required number of crypts at 150 g for 10 min at 4°C.

Methods video S1. Dissection of the intestine (part 1A: isolation of intestinal crypts, steps 1–7 and part 2: isolation and culture of mucosal enteric glial cells , steps 31-37) )

Methods video S2. Scraping the villi (part 1A: isolation of intestinal crypts, step 10)

Methods video S3. Crypt dissociation by mechanical shaking (part 1A: isolation of intestinal crypts, step 16)

#### Part 1B: Crypt culture to generate organoids

25. On ice, mix Matrigel with ENR media at a 70:30 ratio. Use 50 μL of this 70% Matrigel per well.

***Note:*** For a full 24-well plate: 840 μL of Matrigel + 360 μL of ENR media = 1.2 mL total.

***Note:*** Manipulation of Matrigel should always be on ice to avoid polymerization. Pipette the Matrigel slowly to avoid introducing bubbles.

**Figure 2:**
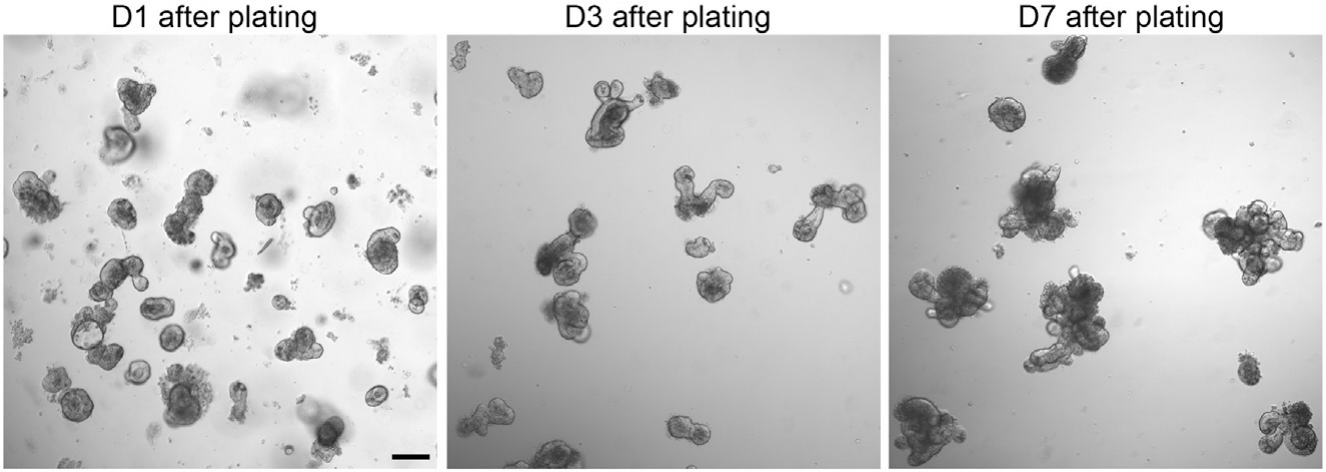
Organoid development at day 1, 3 and 7 after seeding Scale bar, 150 μm.

26. Gently resuspend crypt pellets in the required volume of 70% Matrigel until the clumps become fully dissociated.
27. Remove the 24-well plate from the incubator. Place a 50 μL drop gently in the center of each well.
28. Incubate the plate at 37°C, 5% CO_2_ for 15 min to allow Matrigel polymerization.
29. Add 350 μL of pre-warmed ENR media per well.
30. Culture the organoids 37°C, 5% CO_2_. Replace the media with fresh pre-warmed ENR media every 4 days (Figure 2).

### Part 2: Isolation and culture of mucosal enteric glial cells

**Timing: 3 h**

The current protocol describes the isolation of enteric glial cells from the submucosa by combining EDTA chelation with non-enzymatic dissociation. As submucosal and myenteric EGCs share the same markers, the physical separation of the epithelial mucosa from the smooth muscle was utilized to specifically isolate mucosal EGCs. Although the initial cell suspension consists of 50% mucosal EGCs and 50% other stromal cells, after 3 days in culture, EGCs represent 95% of the cells in culture. This is achieved by culture conditions favorable to EGC growth, which include EGC-friendly substrates (PDL and laminin) and GDNF supplementation (Wang et al., 2018; Baghdadi et al., 2021).

31. Euthanize mice using an authorized, legal method approved by the institution where the research is conducted.
32. Sterilize the abdomen with 70% Ethanol.
33. Make an incision into the abdominal cavity from the external genitalia to the rib cage by cutting abdominal musculature on both sides.
  a. Lift the liver and cut the intestine from the stomach at the pyloric sphincter.
  b. Cut across the large intestine just prior to the anus to release the whole gut.
  c. Cut the mesenteric attachment of the intestine if necessary and gently pull the gut out of the abdominal cavity (Methods video S1A).
34. Place the gut in a petri dish filled with the ice-cold gut buffer.
35. Tear the mesentery to unfold the gut (Methods video S1A).

***Note:*** Make sure to remove any residual mesentery from the tissue.

36. Separate the small intestine from the colon by cutting the gut at the ileocecal junction (Methods video S1A).
37. Flush fecal content from the intestine with the ice-cold gut buffer (Methods video S1B) using a 1 mL syringe mounted with a 1,000 μL tip.
38. Cut the intestine into 3 pieces (the duodenum, jejunum and ileum) and remove the longitudinal muscle.
39. Separate the wooden stick from the cotton tip of a cotton swab. Soak the wooden part of the cotton swab in the cold gut buffer (Methods video S4).
40. Slowly thread one intestinal segment onto the pre-wetted wooden stick (Methods video S4).
41. Use a scalpel or razor blade to make vertical incisions along the intestine and tease away the longitudinal muscle by applying gentle horizontal strokes using the pre-wetted cotton swab (Methods video S4).
42. Sustain this motion until the longitudinal muscle peels away from the intestine. Discard the longitudinal muscle (Methods video S4).

***Note:*** Removing the muscle layer will discard EGCs from the myenteric plexus and avoid their contamination of the mucosal EGC culture.

**CRITICAL:** Incisions should not be too deep to prevent intestinal opening.

43. Cut open the intestinal segment using the forceps/razor blade to release it from the cotton swab (Methods video S4).
44. Keep the intestinal tissue in a 50 mL conical tube filled with the 20 mL gut buffer on ice. Repeat steps 40–43 with the two other intestinal segments.
45. Recover the intestinal segments with forceps and cut them into 0.5 cm pieces.
46. Transfer the pieces to a 50 mL conical tube filled with 25 mL of the ice-cold EGC isolation solution.
47. Gently shake the tube in either an orbital shaker or a rocking plate at 4°C (fridge or cold-room) for 15 min.
48. Triturate the tissue pieces by pipetting up and down 20 times the tissue-solution mixture using a moistened 10 mL plastic pipette.

***Note:*** Moistening the pipette with EGC dissociation solution will avoid tissues sticking inside.

**Figure 3:**
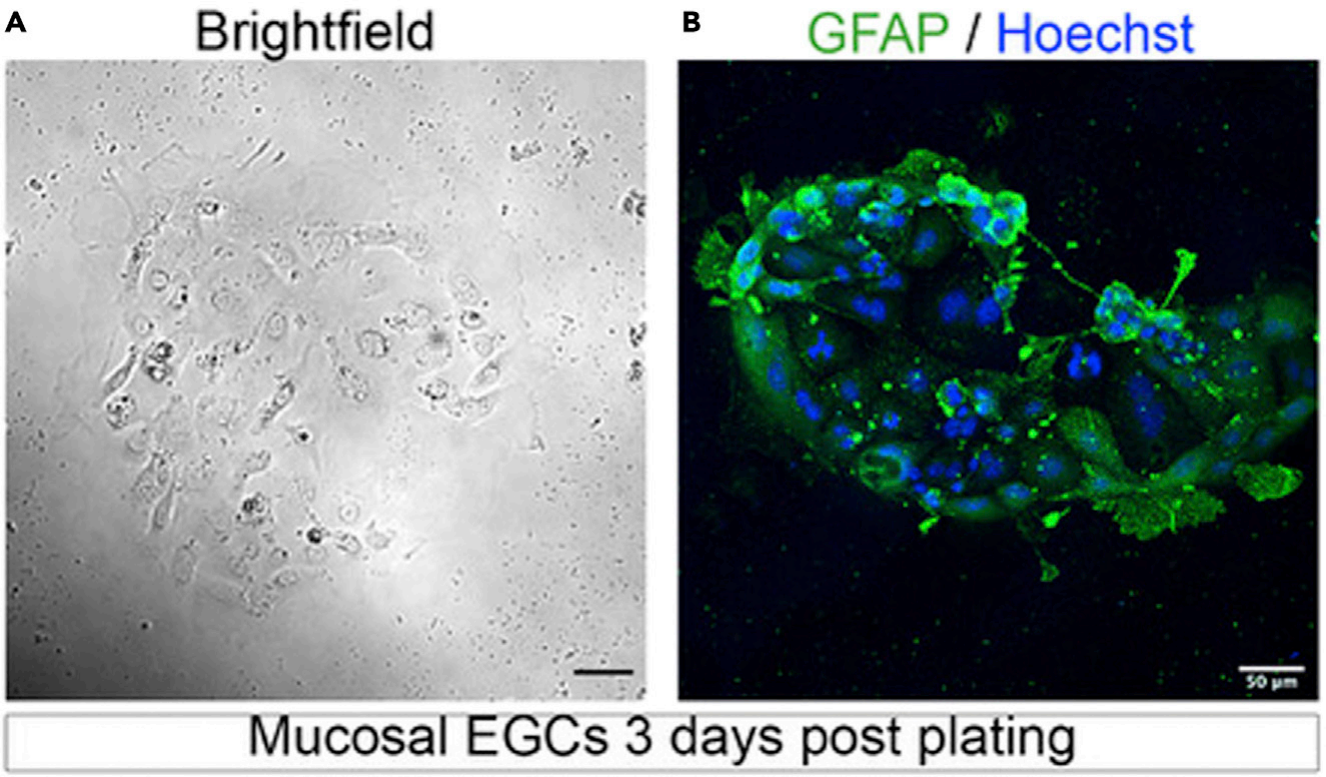
Mucosal EGCs three days after plating (A and B) Successful EGC preparation should form clusters of adherent cells (A), expressing the glial cell marker, GFAP (B). Scale bar, 50 μm.

***Note:*** After 24 h of culture, patches of adherent glial cells are visible by light microscopy (Figure 3A) or immunostaining with specific markers such as GFAP (Figure 3B).

49. Recover the tissues by filtering the mixture through a 100 μm strainer.
50. Use forceps to transfer them to a new 50 mL conical tube filled with 25 mL of the ice-cold EGC isolation solution.
51. Repeat three times, or until the solution is clear.
52. Transfer tissue pieces to a 15 mL conical tube containing 10 mL of the Corning Cell Recovery solution and rock for 30 min at 4°C (cold room or fridge).
53. Triturate the tissue pieces by pipetting up and down 20 times the tissue-solution mixture using a moistened 5 mL plastic pipette to dissociate EGCs from the mucosa.
54. Filter through a 40 μm strainer in a 50 mL conical tube. Discard the tissue pieces.
55. Wash the strainer with 2 mL of cold PBS 1×. Transfer the flow-through to a 15 mL conical tube.
56. Spin at 2,000 g and 4°C for 5 min.
57. Gently resuspend pellets in 1 mL of EGC culture media.
58. Plate 330 μL of cell suspension in a well containing 170 μL of EGC culture media (PDL and laminin coated 12-well plate; 3 wells in total).
59. After 24 h, slowly pipette off the media containing non-adherent stromal cells.
60. Gently rinse with PBS 1×. Replace with fresh pre-warmed EGC culture media.
61. Cells can be used for experiments 3–4 days after plating (Figure 3).

Methods video S4. Mucosal enteric glial cell dissociation (part 2: isolation and culture of mucosal enteric glial cells, steps 39–43)

### Part 3: Organoid culture with enteric glial cell conditioned media

**Timing: 2 h**

**Figure 4:**
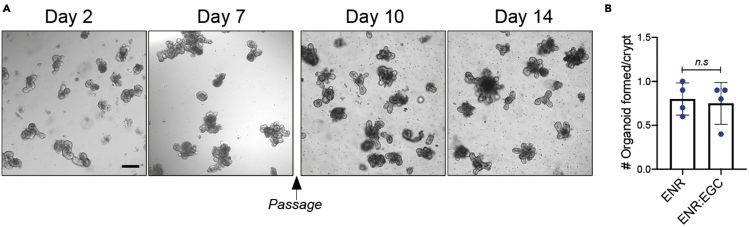
Organoids cultured with EGC conditioned media sustain growth and survival over time (A) Organoids cultured with ENR:EGC-CM (50:50) for 2, 7, 10 and 14 days. Organoids were passaged on day 7 and kept in culture with EGC-CM until analysis. (B) Quantification of the number of organoids formed per crypt 2 days after plating and culturing in EGC-CM. Scale bar, 150 μm. n=4 mice. Error bars indicate SD. n.s: non significant *p* value; two-tailed paired Student’s t test.

Organoids can be combined with conditioned media from primary EGC culture. We previously demonstrated that EGC media alone are not sufficient to sustain organoid growth (Baghdadi et al., 2021). Thus, we mixed 50% of EGC conditioned media (EGC-CM) with 50% of ENR organoid media. In these conditions, organoids can be expanded and passaged as usual for at least two weeks (Figure 4). In addition, EGC-CM can also be used for organoids that have been in culture for several months with the same efficiency.

62. After 3–4 days of culture (following part 2: Isolation and culture of mucosal enteric glial cells, step 61), aspirate the EGC culture media and wash the wells twice with PBS 1×.
63. Add 500 μL of fresh pre-warmed EGC culture media without serum and GDNF.

***Note:*** Removing serum is essential to avoid adding extra growth factors to organoid culture.

64. 24 h later, collect this serum-free EGC conditioned media (EGC-CM) into a 15 mL conical tube. Pool all the wells from one mouse together.
65. Filter the EGC-CM through a syringe equipped with a 0.22 μm pore size filter.

**Pause point:** Filtered EGC-CM can be stored at −80°C for later use and thawed at 37°C for organoid culture.

66. Mix EGC-CM with ENR media at a 50:50 ratio: 125 μL of EGC-CM + 125 μL ENR media per well. Incubate this solution at 37°C for 10 min.
67. 4 days after culture (following part 1: Preparation of crypt organoid culture, step 30), aspirate the ENR media from organoid culture.
68. Add 350 μL the ENR:EGC-CM mix.
69. Incubate the organoids at 37°C and analyze them 48 h–72 h later (see part 4: Analysis of organoids).

### Part 4: Analysis of organoids

One common method to analyze stem cell function using organoids is to measure the efficiency of *de novo* crypt formation (Figure 6A). However, this phenotypic analysis does not allow a deep understanding of the different cell populations present in the crypts. Here, we propose two methods to assess crypt cellular composition and stem cell properties: a) RT-qPCR for transcriptional analysis (part 4A. RNA extraction for transcriptional analysis by RT-qPCR) and b) flow cytometry for analysis of the intestinal stem cell (ISC) number (part 4B: Cellular analysis of organoids by flow cytometry).

#### Part 4A. RNA extraction for transcriptional analysis by RT-qPCR

**Timing: 7–8 h**

**CRITICAL:** RNA molecules are highly sensitive to degradation. To isolate intact, high-quality RNA, it is essential that RNases are not introduced throughout the RNA extraction procedure. Decontaminate pipettors, benchtops, centrifuge, and ice bucket with a surface decontamination solution like RNAse Away®, and use RNAse-free tips, tubes, PBS 1× and H_2_0. In addition, the whole procedure should be done fast to avoid RNA degradation.

70. Following step 69, remove media from organoid culture and add 400 μL of ice-cold PBS 1× per well. Incubate the plate on ice for 5 min.
71. Break up the Matrigel-organoid dome by pipetting up and down.

***Note:*** To avoid bubbles, set the pipette at 250 μL.

72. Pool two wells into a 15 mL conical tube filled with 5 mL of ice-cold PBS 1×. Pipette up and down with a 1,000 μL pipette 15 times to further dissociate the Matrigel.

***Note:*** For solid statistical analysis, we recommend to pool 2 wells for one qPCR data point and use 3 data points per mouse and/or condition.

73. Centrifuge at 150 g for 10 min at 4°C.
74. Carefully discard the supernatant and resuspend the pellet in 350 μL of Qiagen RLT buffer supplemented with 1% β-mercaptoethanol.

**CRITICAL:** β-mercaptoethanol is toxic and should be used in the fume hood.

75. Vortex the tubes for 30 s to fully lyse the organoids.
76. Spin the tubes at 150 g for 1 min at 4°C.

**Pause point:** At this step, the lysate can be transferred to a RNAse-free 1.5 mL tube, snap frozen in dry ice and kept at −80°C until ready for RNA extraction.

77. Proceed with the Qiagen RNeasy® Plus Mini extraction kit according to the manufacturers’ instructions: https://www.qiagen.com/us/resources/download.aspx?id=14e7cf6e-521a-4cf7-8cbc-bf9f6fa33e24&lang=en.

**Pause point:** RNA can be kept at −80°C until ready for the reverse transcription step.

78. Measure RNA yield and concentration with a Nanodrop spectrophotometer.
  a. For an optimal amplification, use ≤ 50 ng of RNA for one qPCR well.
  b. Transfer the necessary quantity of RNA in a clean RNAse-free 1.5 mL tube.
  c. Perform reverse transcription according to manufacturers’ guidelines: https://www.thermofisher.com/document-connect/document-connect.html?url=https%3A%2F%2Fassets.thermofisher.com%2FTFS-Assets%2FLSG%2Fmanuals%2FsuperscriptIII_man.pdf.

**Pause point:** cDNA can be stored at −80°C until ready to load qPCR plate.

79. Run qPCR following manufacturers’ instructions: https://www.thermofisher.com/document-connect/document-connect.html?url=https%3A%2F%2Fassets.thermofisher.com%2FTFS-Assets%2FLSG%2Fmanuals%2Fcms_042179.pdf.

***Note:*** For 1 qPCR well: Mix 5 μL of SYBR green master mix + 4 μL cDNA + 1 μL primer mix (forward + reverse at 10 μM).

**CRITICAL:** Load qPCR plate on ice.

80. Analyze data using the 2^-ΔΔCT^ method (Livak and Schmittgen, 2001). We recommend the use of two independent genes for expression normalization. First normalize the gene of interest with each housekeeping gene separately and perform the mean value of both to obtain the final 2^-ΔΔCT^ value.

***Note:*** Specific forward and reverse primers for commonly used crypt population markers are listed in Table 1. Tbp and Rpl13 are normalization housekeeping genes.

#### Part 4B: Cellular analysis of organoids by flow cytometry

**Timing: 3 h**

**Figure 5:**
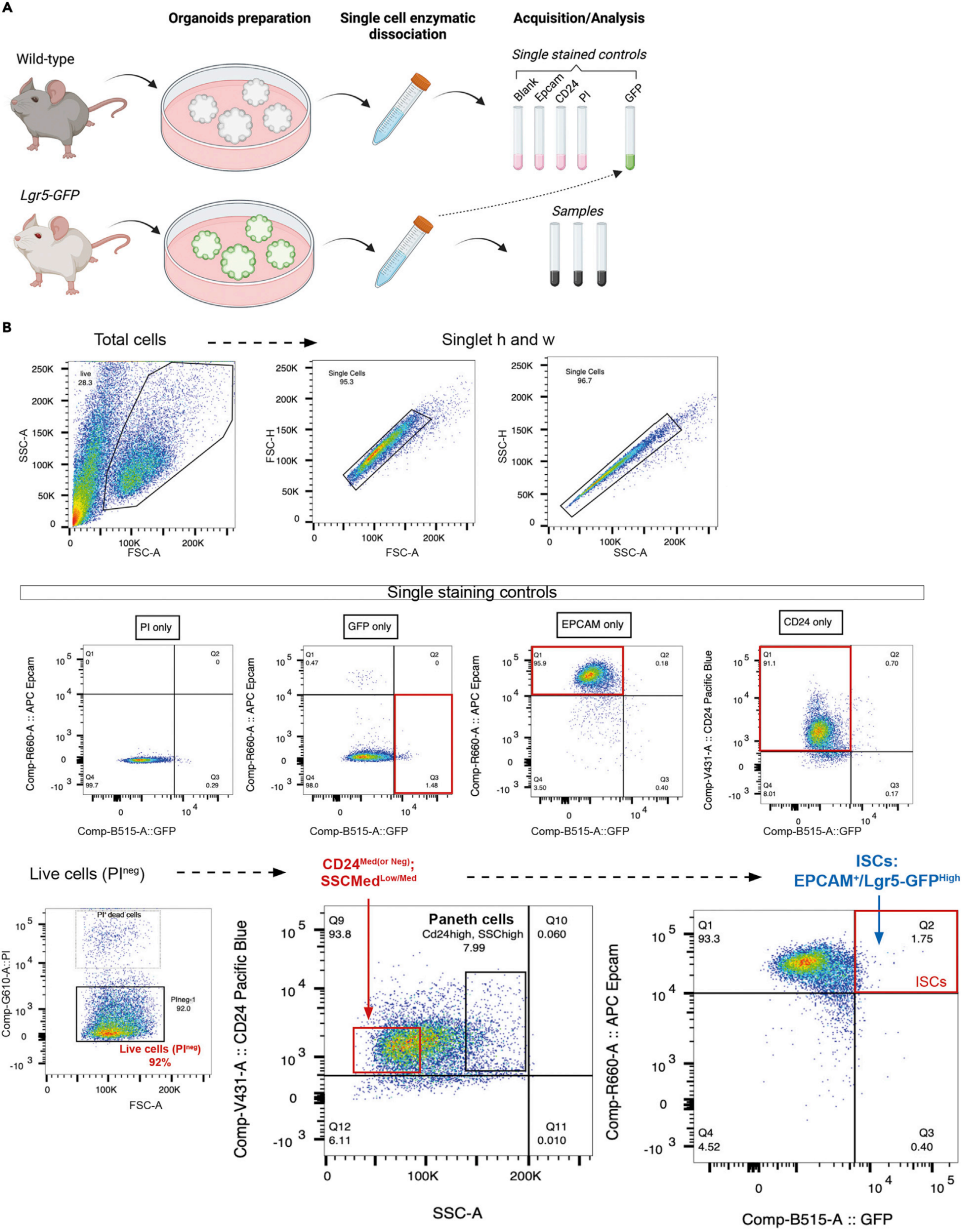
Analysis of Lgr5^High^ ISC frequency in organoids by flow cytometry (A) Representative scheme of the experimental flow. (B) Gating strategy to determine ISC proportion in organoids. First, doublets are gated out by FSC-H (forward scatter) versus FSC-A and then by SSC-H (side scatter) versus SSC-A gates. Second, single staining controls of PI, GFP, Epcam-APC and CD24-Pacific Blue are performed to distinguish the positive signal from the background negative signal and to evaluate if fluorescence compensation is required. After setting up all parameters (laser, compensation, positive signal), organoid samples can be analyzed. After gating on singlet, dead cells are excluded with Propidium Iodide (PI) staining. CD24 is plotted versus SSC and cells with low/medium expression of CD24 (20% of total CD24^+^ cells), and low granularity (40% of SSC) labeled as CD24^Low/Med^ /SSC^Low/Med^ are selected. From this cell population, Epcam-APC is gated versus GFP, and all the Epcam^+^/Lgr5-GFP^High^ cells, labeled as ISCs, are selected. FACS plots were originally depicted in Baghdadi et al. (2021).

For analysis of ISCs by flow cytometry, we use crypt organoids derived from *Lgr5-GFP* mice (Barker et al., 2007) for labeling ISCs and organoids from wild-type (no GFP) mice for setting up the negative staining and single stained controls (Figure 5A).

81. Pre-warm 5 mL of TrypLe 1× at 37°C.
82. Following step 69, remove media from organoid culture and add 400 μL of the ice-cold PBS 1X per well. Incubate the plate on ice for 5 min.
83. Break up the Matrigel-organoid dome by pipetting up and down.

***Note:*** To avoid bubbles, set the pipette at 250 μL.

84. Pool all the wells for one mouse/condition into a 15 mL conical tube filled with 3 mL of ice-cold PBS 1×. Pipette up and down 15 times to further dissociate the Matrigel.

***Note:*** Because cells will be lost during the washing steps and enzymatical dissociation, we recommend starting with minimum 6 wells of organoids for one data point.

85. Centrifuge at 150 g for 10 min at 4°C.
86. Carefully discard the supernatant and resuspend the pellet in 5 mL of pre-warmed TrypLe 1×.
87. Incubate the suspension at 37°C for 3 min.
88. Further dissociate organoids into single cells by mechanical trituration using a 5 mL syringe equipped with a 18G blunt needle. Pass the suspension 15 times through the needle.
89. Neutralize the TrypLe by adding 10 mL of the cold gut buffer.
90. Spin 400 g, 5 min at 4°C.
91. Resuspend the pellet in 500 μL of the gut buffer.
92. Add surface marker antibodies (see below) and incubate for 20 min on ice in the dark or by covering the ice bucket with an aluminum sheet.
  a. CD24 antibody conjugated with Pacific Blue (CD24-Pacific blue): 1/500 = 1 μL in 500 μL of sample.
  b. EPCAM antibody conjugated with APC (EPCAM-APC): 1/500 = 1 μL in 500 μL of sample.
  c. Propidium iodide (live dye to exclude dead cells): 1/1000 = 0.5 μL in 500 μL of sample.

***Note:*** EPCAM is used to mark all epithelial cells and CD24 to differentiate ISCs (CD24^Neg^) and Paneth cells (CD24^High^). CD24 and EPCAM can be conjugated to different fluorochromes as long as excitation and emission curves are compatible with each other and endogenous Lgr5-GFP. For fluorochrome validation and compatibility, use the BD spectrum viewer.

**CRITICAL:** Set aside 50 μL of control sample before adding antibodies: it will be used as a GFP single stained control to set up the cytometer parameters.

93. Add 5 mL of the gut buffer to wash off the excess antibody.
94. Spin at 400 g for 5 min at 4°C.
95. Discard the supernatant. Resuspend the pellet in 500 μL of the gut buffer.
96. Proceed to flow cytometry acquisition and analysis (Figure 5B).

## Expected outcomes

**Figure 6:**
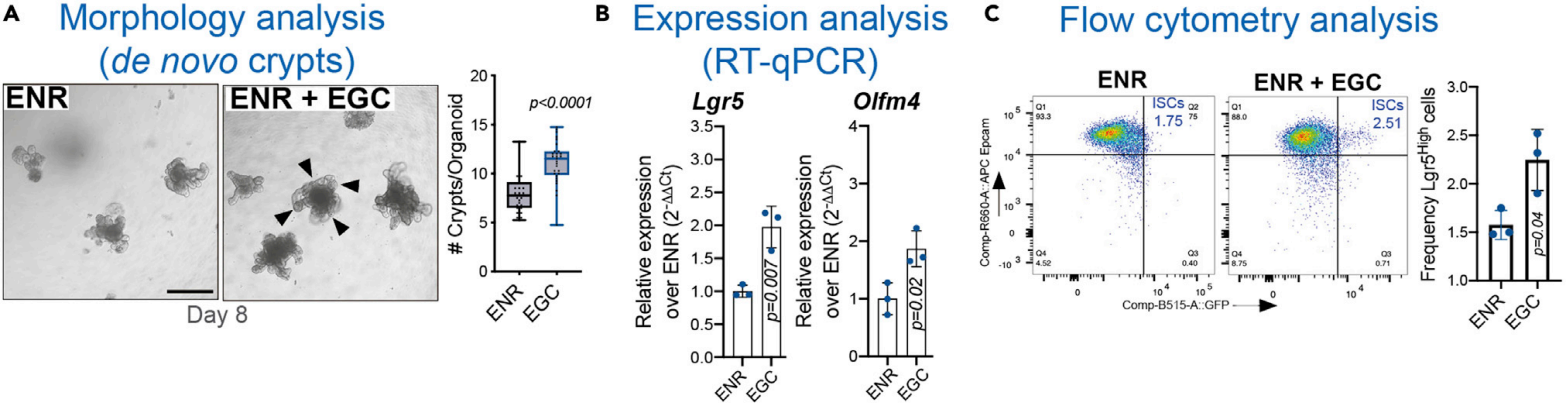
Expected outcomes (A–C) Conditioned media from mucosal EGC culture increases (A) the number of crypts per organoid, (B) the expression of stem cell markers (*Lgr5*, *Olfm4*) and (C) the frequency of Lgr5^High^ stem cells. FACS plots were originally depicted in Baghdadi et al. (2021). Scale bar, 150 μm. n=4 mice. *p* values are shown in the panels. Error bars indicate SD; Wilcoxon test (A); two-tailed paired Student’s t test (B, C).

This protocol for 3D organoid culture combined with conditioned media isolated from primary EGC culture was developed to investigate the mechanisms of intestinal stem cell interaction with mucosal EGCs. RT-qPCR, flow cytometry and morphological analyses will provide information regarding stem cell numbers, expression levels of specific markers, and organoid size and shape*.* The experiments can be stopped at different days of organoid culture to observe the dynamics of crypt cells responding to EGC niche signals over time. It is expected that the combination of ENR with EGC conditioned media significantly promotes *de novo* crypt formation (Figure 6A), while increasing expression levels of stem cell markers (*Lgr5*, *Olfm4*) (Figure 6B) and the number of ISCs with high *Lgr5* expression (Lgfr5^High^ ISCs) (Figure 6C). Altogether, these results indicate enhanced stem cell activity.

## Limitations

This protocol describes an organoid culture system combined with primary mucosal EGC conditioned media. However, because organoids and EGCs are not in direct contact, it can only be utilized to study their interaction through secreted factors. Therefore, the potential physical EGC-organoid interactions cannot be analyzed in this system.

As mentioned above, mucosal and myenteric EGCs share the same markers. Consequently, the presence of glial cells from the inter-myenteric plexus cannot be excluded.

Another limiting factor is the cost of Matrigel and recombinant proteins needed for technical and biological replicates. To reduce this cost, Matrigel can be diluted down to 1:1 in ENR (instead of 70:30). Another approach could be to scale down the organoid cultures from a 24 to a 48-well plate and use 30 μL Matrigel dome and 200 μL ENR medium per well.

Finally, we expect to isolate 50,000–100,000 EGCs from one whole mouse intestine. Thus, crypts and EGCs cannot be isolated from the same mouse and the experiment requires the use of multiple mice.

## Troubleshooting

### Problem 1

Low crypt yield (part 1A: Isolation of intestinal crypts, step 22).

### Potential solution

Check if crypts have been lost in the first fraction upon chelation. If yes, decrease the EDTA concentration of the chelating buffer to 2 mM. If crypts are not lost at the chelation step, they might still be attached to the tissue during the dissociation step. Increase the shaking time up to 8 min and verify under the microscope if crypts are detached before proceeding to the next steps of the protocol.

### Problem 2

EGCs do not adhere or detach (part 2: Isolation and culture of mucosal enteric glial cells, step 59).

### Potential solution

Use fresh laminin and PDL to coat plates. After plating the cell suspension, do not disturb the culture plate for 24 h.

### Problem 3

Low RNA yield and/or quality after isolation from organoids (part 4A. RNA extraction for transcriptional analysis by RT-qPCR, step 78).

### Potential solution

This is likely due to the presence of residual Matrigel around the organoids, which might prevent successful cellular lysis. Another way to break the organoid dome is to pass the suspension 15 times through a 18G blunt needle attached to a 5 mL syringe (part 4A. RNA extraction for transcriptional analysis by RT-qPCR, step 71). Moreover, the use of a specific buffer such as the Cell Recovery Solution® can also help to break down the Matrigel (part 4A. RNA extraction for transcriptional analysis by RT-qPCR, step 70). If the quality of RNA is good but the quantity is low, increase the number of wells per sample. Another possibility is to purify RNA using the Qiagen RNAeasy Micro kit that has an elution volume of 15–25 μL (instead of 35–50 μL for the Qiagen RNAeasy Micro kit). This will increase RNA concentration and yield.

### Problem 4

Low number of cells for flow cytometry analysis (part 4B: Cellular analysis of organoids by flow cytometry, step 96).

### Potential solution

In this part of the protocol, cell pellets are small, so they might not be easily visible. When aspirating the supernatant, leave some liquid (about 500 μL) at the bottom of the tube to make sure the cell pellet is not lost. Another possibility is to increase the number of wells per sample/data point.

### Problem 5

Crypts sink and stick at the bottom of the well (part 1B: Crypt culture to generate organoids, step 28).

### Potential solution

During Matrigel polymerization, crypts can sink at the bottom of the well and stick to the plastic. To avoid this problem, turn the plate upside down during Matrigel polymerization so they can form hanging droplets.

## Resource availability

### Lead contact

Further information and requests for resources and reagents should be directed to and will be fulfilled by the lead contact, Tae-Hee Kim (tae-hee.kim@sickkids.ca).

### Materials availability

This study did not generate new unique reagents.

## Data Availability

This study did not generate datasets.
